# Marion Julia Lamb (29 July 1939–12 December 2021)

**DOI:** 10.1093/eep/dvac009

**Published:** 2022-03-16

**Authors:** Eva Jablonka

**Affiliations:** Cohn Institute, Tel-Aviv University, Tel Aviv 69978, Israel

Marion Julia Lamb, a pioneer in the field of evolutionary epigenetics, died in London on the 12th of December 2021 at the age of 82 of lung cancer.

Marion was an original and accomplished scientist and her intellectual brilliance was combined with deep political and intellectual courage, a fascination with the natural world and an almost fanatical studiousness. Coming from a nature- and book-loving working-class family, she roamed, as a child, the coasts and estuaries of East Anglia, watching birds, investigating rock pools, turning every rotten log, developing the naturalist’s ardent and focused competence. She was always grateful to her parents for the freedom they gave her and for their one demand—that she ‘does her best’—whatever ‘best’ may be. And indeed she did—from decorating her flat to gardening, sailing, teaching and researching. Her intellect was clear and powerful and she excelled in everything she ever put it to—as a 16-year-old lab assistant in Max Perutz’s lab in Cambridge during her high-school vacations, as a brilliant university student (she shared with Robin Weiss the Francis Perch Bedford Prize for the best first degree in University College London), as an inspiring teacher and as a ground-breaking scientist.

Marion loved the elegant beauty of genetics, and when John Maynard Smith, her genetics teacher in University College London (UCL) suggested that she does a PhD with him, she was delighted. Her thesis on ‘Radiation and Ageing in *Drosophila*’ was awarded a PhD in 1965. Her laboratory research was conducted in UCL, Harwell and Birkbeck College (where she became a senior lecturer) and was concerned mainly with various aspects of the biology and genetics of ageing, using *Drosophila* as a research tool. Her large body of experimental work on ageing, radiation biology and mutagenesis, 25 papers altogether, stood the test of time, and she wrote a highly acclaimed, crystal-clear and concise book ‘*The Biology of Ageing*’ (published by Blackie, 1), on which several advanced courses in the biology of ageing around the world were based.

Evolutionary biology was Marion’s passion and guide since she was a high-school student and read Huxley’s *Evolution: The Modern Synthesis*. She told me that the first tutorial she ever attended as a first-year student in UCL was on Waddington’s *The Strategy of the Genes* and that it blew her mind. Our first conversation, in 1973, also happened to be about Waddington (I discovered Waddington, independently, through reading Arthur Koestler’s *Ghost in the Machine,* well before I knew any genetics). I was a first-year student, and she was my genetics teacher in Birkbeck College, where I spent a year. I asked her if she knows Waddington and she looked at me with a wry smile and suggested that I learn to walk before I start running. I ended up doing a PhD in genetics.

Long before we started writing papers together, Marion sent me evolutionary biology books to Israel, and when we met we discussed the many hot topics of the time—punctuated equilibria, the sociobiology debate, the selfish gene and the neutralist-selection debate. We started working together years later, in the early 1980s, exploring the evolutionary implications of epigenetic inheritance. This was not a mainstream topic (to put it mildly) and our interest in it had something to do with our background—Marion was educated in the school of British evolutionary biology, which was, in the 1950s and the early 1960s, far more open to the possibility of unorthodox modes of heredity and evolution than the American counterpart, and I came to biology because of my interest in philosophy and the great debates surrounding evolutionary theory. Our more direct motivations were related to the experimental work in genetics and chromatin biology that we were doing at the time.

In late 1982, I started a PhD in the Genetics department of the Hebrew University on the relationship between DNA methylation and time of chromosomal replication. I used female cell lines where the two X chromosomes could be morphologically distinguished and asked whether the inactive X chromosome can alter its inactive, condensed chromatin conformation and its late time of replication when the cells were treated with a demethylation agent, 5-azacytidine. The answer was positive, but the chromosome‐wide effect that I found was transient. This suggested that the dynamics of DNA methylation and chromatin changes are more flexible than hitherto thought. Marion was investigating at that time the effects of ageing on polytene chromosomes in *Drosophila* and found that chromatin structure was changed with age (unfortunately, she never published these results). We thought that the mix of stable transmissibility of chromatin states in cell lineages on the one hand and the developmental responsiveness of these states on the other open up very intriguing evolutionary questions and possibilities. We argued that it was implausible that all traces of past-induced chromatin variations would become deleted during gametogenesis. As long as totipotency is maintained, chromatin variations, just like genetic variations, could be inherited through the germ line. We reasoned that since chromatin states can be environmentally induced, chromatin variations acquired during development may be passed on between generations.

Since our framework was evolutionary, we decided to look at the dynamics of X chromosome activation and inactivation during development and evolution. We focused on the developmental effects of meiotic pairing on chromatin organization and asked how chromosomal developmental dynamics affected the evolution of sex chromosomes. These investigations yielded two papers. Our first joint published paper was ‘Meiotic pairing constraints and the activity of sex chromosomes’ [2] (completed in 1986 but published in 1988 after much toing and froing) and the second paper, ‘The evolution of heteromorphic sex chromosomes’ which [4] we regarded as our most heroic effort, took 4 years of research and was published in 1990 [3]. In parallel, we were writing what we regarded as our programmatic paper about the role of epigenetic inheritance in evolution ‘the evolution of acquired epigenetic variations’ [3]. This paper, which is now regarded as a classic (it was re-published and commented on in 2015, 19), was published in 1989 in the *Journal of Theoretical Biology* (*JTB*) after being rejected editorially by both *Nature* and *Science.* It was eventually published because of the providential intervention of John Maynard Smith. Marion met John in the 1988 Genetics Society meeting, just before he went for a few months to Princeton, and they exchanged the typical academic gossip. He asked her what she was working on and she told him about the paper we were trying, unsuccessfully, to publish. John was intrigued (he had a very high opinion of Marion’s ability, after working and publishing papers with her) and asked her to send him the rejected paper. Although he disagreed with our conclusions he thought the paper was important and wrote to Lewis Wolpert, who was the editor of *JTB* at the time, urging him to have a look at it. In this paper, we suggested that the between-generation inheritance of developmentally and environmentally induced epigenetic variations can be explained on the basis of already partially characterized cell-memory epigenetic mechanisms and that this kind of heredity has important evolutionary implications, making it possible to explain ‘soft’ or ‘Lamarckian’ inheritance.

In the ensuing years we worked intensely on the evolutionary implications of epigenetic inheritance. We reasoned that since genomic imprinting showed that epigenetic changes occur in gametogenesis and are transmitted to zygotes, and since heritable variations in chromatin structures may have multiple selective advantages that go beyond the specific advantages of genomic imprinting, we are bound to find studies in the classical genetics literature that can be best interpreted in terms of such inheritance. (We found, indeed, many such examples.) We published papers on epigenetic inheritance and ageing [5, 8], on epigenetic inheritance and speciation [6, a paper that took 2 years to publish], on the mechanisms of epigenetic inheritance and on models of gametic transmissibility of induced epigenetic variations [7] and eventually our first book, which put all our ideas together: *Epigenetic inheritance and Evolution: The Lamarckian Dimension* [10]. We started writing the book in 1990, and it was published in 1995 by Oxford University Press (John Maynard Smith was one of their reviewers and vouched for our sanity). We decided to write a book because we became tired of the constant struggles with journal editors and journal reviewers, of writing papers which present only limited aspects of the subject and required uneasy compromises. We believed that a systematic synthesis of the literature on epigenetic inheritance and a systematic exploration of the effects of epigenetic inheritance in evolution are needed if evolutionary epigenetics is to get off the ground. The subtitle of our book, *The Lamarckian Dimension*, raised many hackles, but we decided to acknowledge the evolutionary tradition of Lamarck as well as that of Darwin. Both their portraits appear on the book’s cover.

These were exciting times. The technologies of molecular biology were developing, and the initially meagre molecular evidence for between-generational epigenetic inheritance was rapidly growing. In 1994–95, I spent a happy period in the Collegium Budapest with the Theoretical Biology Group assembled by Eörs Szathmáry. This led to collaborative work on several different topics, with one of the outcomes being the development of theoretical models [11, 12] that explored some of the conditions under which epigenetic inheritance could have evolved. John Maynard Smith and Marion visited the group several times and the liberating effect of excellent Hungarian wines, friendship and the love of science and ideas led to some of the best discussions I have ever participated in—on anything and everything in biology. However, not all the reactions that Marion and I faced were constructive and fair. Some of the opposition we faced was aggressive, patronizing and even abusive. Unlike me, Marion, who was the greatest critic of our work, was nonplussed. ‘It is their problem, Eva,’ she used to tell me when I got upset by these reactions. On the whole, however, we were very happy. When Patrick Bateson asked me, many years later, what it was like to be a lone voice in the desert, I said that I was not alone, Marion and I had each other’s critical and supportive company and the desert was nice, quiet and offered much freedom.

Our work on epigenetic inheritance turned Marion into a historian of biology and made her interested in the philosophy of science. She became intrigued and fascinated by the turns and twists in the history of ideas, in the history of misconceptions and dogmas, in the ways in which ‘common knowledge’ (such as the non-inheritance of acquired characters) was formed, in the role of politics and the use of words, the subtle ways in which they shaped thought [13, 15–17]. One of the studies she left unfinished when she died was a big book on the history of heredity, which got stuck in the thirteenth century with Albertus Magnus about whom she kept reading with ever-growing admiration.

Marion is probably best known for *Evolution in Four Dimensions* (E4D) [14], which was published in 2005, and translated into eight languages. We started writing it in 2000 but we discussed the notion that concepts of heredity and evolution need to be expanded and that Neo-Darwinism had outlived its usefulness much earlier. In 1994 I managed to publish a paper (which was mouldering in editors’ offices for 3 years) entitled ‘Inheritance systems and the evolution of new levels of individuality’, [9]. The main topic of that paper, which was influenced by Leo Buss’ 1987 book *The Evolution of Individuality* and which ended up being published in old, faithful *JTB*, was the role of epigenetic inheritance in the evolution of multicellularity, but the role of language in the evolutionary construction of human social groups was also discussed. Moreover, since 1992 I was working on the role of social learning in animal evolution with behavioural ecologist Eytan Avital, and since 1996 on the evolution of the linguistic capacity with linguist Daniel Dor. Although Marion was not a co-author on these publications, she was deeply involved in every aspect of the work and nothing was ever published before it was scrutinized and approved by her. These studies and the persistent pressure from our friends and colleagues that we write a book, which will be broader and more widely accessible than our 1995 book, led us, after much trepidation, to embark on an integrative new synthesis of evolution from an extended heredity perspective. E4D was certainly more widely read than our 1995 book, and before and since its publication we wrote numerous articles which summarized, refined, extended and historicized our views, wrote a second revised and extended edition [18] and published a short book on inheritance systems and the extended evolutionary synthesis [20]. I believe that this corpus of work contributes to the integrative evolutionary-developmental synthesis that is under construction today, opposed by an ever-shrinking community of mostly elderly population geneticists.

**Figure:**
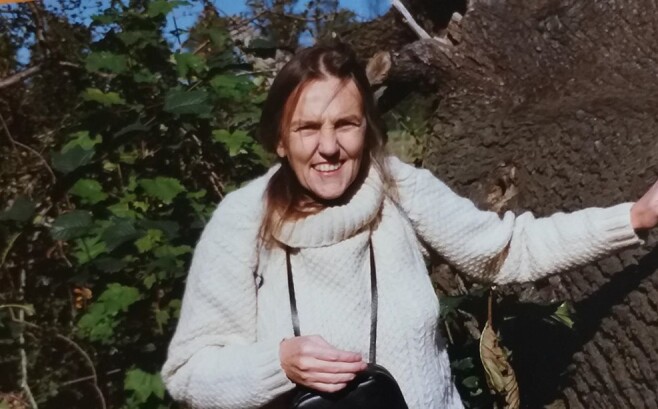
**Photo**: Marion J. Lamb, 1986, never separated from her binoculars. Trent Park, London. Credit: Revital Katznelson

Marion was a formidable teacher, and her students were in awe of her, admired her and tried to live up to her very exacting standards. She was a naturalist who travelled widely, a theatre addict who took full advantage of what the London theatre scene had to offer and an intellectual with an astounding knowledge of architecture, the history of biology, literature and classical music—although only close friends realized the depth and scope of her knowledge and scholarship. Although she rarely talked about her own work and about her own passions, she did express, eloquently and without compromise, her opinions on matters of academic and intellectual integrity and freedom, on politics and social justice, and always took action. She despised pretentiousness, sloppiness and dogmatism. Race, class and gender inequality infuriated her. She loathed what she called ‘the little academic games’ of so many of her colleagues and never participated in them. She wanted no special honours and no special awards. I know that she would have been uncomfortable; in fact she would have been quite appalled, by this obituary, telling me to stop wasting people’s time and get on with my work. She never took what she regarded as undeserved credit, and although very often she made substantial contributions to her colleagues’ work she flatly refused their offers to be a co-author.

Marion fought for what she believed others needed and deserved openly and with a fierceness that was sometimes seen as ruthless and treated social injustice and political oppression with the same uncompromising determination. Her colleagues and her students knew that they can rely on her, that she will fight for them and with them if she believed that their cause—political, social or intellectual—was just. ‘Ferocious and incredibly kind’ is how her colleague, the late palaeontologist John Attridge, described her. Another colleague compared her to a lighthouse. She was a moral compass for her friends and family and supported them in every conceivable way, and she devoted endless time and hard work to improve the manuscripts her colleagues kept sending her to read and comment on. Simona Ginsburg, one of her friends and colleagues described her thus: ‘In her research, Marion was unsurpassable in perspective, sheer hard work, and detail. Her writing was crystal-clear and her arguments original and impeccable. She was generous as a colleague and offered constructive criticism, always delivered with a sense of humor, well documented references, corrections and insights that improved one’s own scholarship and made one’s own writing much more elegant’.

Marion was a role model for many people. Her lasting contribution to science is a testimony not only to her excellence as a scientist but also to her personal courage and determination. Her beautiful clear mind, her total integrity and honesty and her modesty and generosity had a deep effect on the lives and work of those lucky enough to have known her.
